# Effectiveness of polymyxin B hemoperfusion for sepsis depends on the baseline SOFA score: a nationwide observational study

**DOI:** 10.1186/s13613-021-00928-z

**Published:** 2021-09-26

**Authors:** Kenji Fujimori, Kunio Tarasawa, Kiyohide Fushimi

**Affiliations:** 1 Department of Health Administration and Policy, Tohoku University Graduate School of Medicine, Sendai, Japan; 2 Department of Health Policy and Informatics, Tokyo Medical and Dental University Graduate School of Medical and Dental Sciences, Bunkyo-ku, Tokyo, Japan

**Keywords:** Polymyxin B hemoperfusion, PMX, Sepsis, DPC database, SOFA score, Propensity score matching

## Abstract

**Background:**

Polymyxin B hemoperfusion (PMX) aims to treat septic shock by removing endotoxin from the patient’s blood. However, the relationship between the severity of the patient's organ damage and the survival benefit of PMX treatment is not clear.

**Methods:**

We analyzed the efficacy of PMX on adult sepsis patients using the propensity score matching method and the Japanese Diagnosis Procedure Combination (DPC) national inpatient database from April 2018 to March 2020. We stratified the patients into five categories based on their baseline Sequential Organ Failure Assessment (SOFA) score and compared the mortality between PMX-treated and non-treated groups in each category. We also compared continuous hemodiafiltration (CHDF)-, ventilator- and noradrenaline-free days between the groups.

**Results:**

Of 44,177 patients included in the study, 2191 received PMX. After 1:1 propensity score matching, we created matched cohorts of 2033 pairs. PMX significantly improved the survival of the patients in the SOFA score categories of 7–9 and 10–12. On the other hand, there was no significant difference in the survival rate in SOFA score categories of 0–6, 13–15, and 16–24. In analyzing organ support-free days, PMX was also beneficial in the 7–9 and 10–12 SOFA categories compared to other categories.

**Conclusion:**

Analysis of a large-scale Japanese inpatient database found a significant association between PMX efficacy and baseline SOFA score. This result indicates higher efficacy in patients with medium SOFA scores in the range of 7–12. The result provides a promising hypothesis for selecting appropriate patients for PMX and should be validated in future RCTs.

**Supplementary Information:**

The online version contains supplementary material available at 10.1186/s13613-021-00928-z.

## Background

Sepsis is the most common cause of death in the ICU. Sepsis-related deaths were reported as 11 million per year, representing 19.7% of global deaths [1]. In particular, when the disease progresses to septic shock with circulatory failure, the mortality rate is as high as 19–31% [2–4].

The standard treatment for sepsis includes early administration of antimicrobial agents, removal of the infected foci, and early infusion of fluids and vasopressors in case of shock. Also, controlled clinical trials of various adjunctive medications and therapies did not show a clear survival benefit.

One of the adjunctive therapies for septic shock is polymyxin B hemoperfusion (PMX). This therapy uses a polymyxin B-immobilized fiber column to remove endotoxin from the bloodstream [5, 6]. Many studies determined the efficacy of PMX in improving blood pressure and respiratory function. On the other hand, randomized controlled trials (RCTs) of PMX using survival rate as an endpoint have shown mixed results. Some show mortality reduction, and others show no effect [7–9].

RCTs are the means of clinical research that provide the highest level of evidence regarding the efficacy of treatments. However, it is difficult to include many patients affected by acute and severe conditions such as sepsis into the studies in a limited period. Furthermore, RCTs include patients who meet specific criteria defined for each study, which do not always reflect the effectiveness in actual clinical practice. In recent years, studies using real-world big data have become a common alternative to RCTs. One big data source available in Japan is the Diagnosis Procedure Combination (DPC) database [10, 11]. The DPC database contains more than seven million cases per year, collected from more than 1100 facilities across Japan. The data reflect the actual clinical practice in the country.

The DPC data provide information on the diagnosis and treatments performed. Still, it does not provide data on various laboratory values. Thus, it has the limitation of not fully grasping the severity of the diseases. Since April 2018, patients diagnosed with sepsis are required to record their Sequential Organ Failure Assessment (SOFA) score on the day of sepsis diagnosis and the following day in their DPC data. This SOFA score will enable more precise analysis based on the severity of patients' organ damage, which has not been possible in the past.

In this study, we examined the association between SOFA score at the onset of sepsis and the efficacy of PMX using 2 years of DPC data after April 2018.

## Methods

### Study design and data source

This retrospective observational study analyzed the inpatient data from the Japanese Diagnosis Procedure Combination (DPC) database. We extracted the patient data from April 2018 to March 2020, including patients whose primary diagnosis was sepsis based on the ICD-10 codes. We excluded patients who were under the age of 20, whose SOFA score data were missing, who died within 3 days after sepsis diagnosis, who received their first PMX treatment other than on the first or second day of sepsis diagnosis, who were on chronic hemodialysis before sepsis onset, and who transferred to other hospitals within 28 days without improvement. We defined the first SOFA score record as the first day of sepsis diagnosis (day 1). We categorized patients who received PMX on the first or second day of sepsis diagnosis into the PMX group and patients who did not receive PMX into the control group. In addition, we collected baseline information of the patients, such as age at admission, gender, emergency versus elective hospital admission, university hospital or non-university hospitals, admission to emergency rooms or intensive care unit (ICU), and the Charlson Comorbidity Index (CCI) [12, 13]. We also identified the following treatments performed on the first or second day of sepsis diagnosis: continuous hemodiafiltration (CHDF), hemodialysis (H.D.), mechanical ventilation, surgery, administration of γ-globulin, antithrombotic drugs (antithrombin III (AT III) and recombinant soluble thrombomodulin (rTM)), steroid, red blood cell (RBC) transfusion, platelet transfusion, PMX, the maximum daily dose of noradrenaline and SOFA score of the first day. We defined surgery as a surgical operation performed on the day of sepsis diagnosis or within 7 days before the sepsis diagnosis. We did not include emergency treatment procedures such as cardiopulmonary bypass, balloon pumping, tracheotomy, and transfusion in surgery.

### Propensity score matching

We performed a propensity score matching analysis between PMX-treated (PMX group) and non-treated (control group). We estimated the propensity score using a logistic regression model for the use of PMX as a function of the following confounders: the age at admission, gender, emergency versus elective hospital admission, university hospital or non-university hospitals, admission to the emergency room (E.R.) or intensive care unit (ICU) and CCI, CHDF, H.D., mechanical ventilation, surgery, administration of γ-globulin, AT III, rTM, steroid, red blood cell transfusion, platelet transfusion, and the maximum daily dose of noradrenaline. A one-to-one matched analysis using the nearest-neighbor matching was performed based on the estimated propensity score of each patient. We used a caliper width of 0.2 of the standard deviation of the propensity score. We evaluated the balance among covariates using absolute standardized difference (ASD).

### Outcomes

Patients were stratified based on the SOFA scores in the matched population into five categories (SOFA 0–6, 7–9, 10–12, 13–15, and 16–24). We examined the difference in the survival curves and 28-day mortality between the PMX and control groups. We also analyzed CHDF-, mechanical ventilation- and noradrenalin-free days at 28 days in patients on each treatment on day one or day two of sepsis diagnosis. We divided patients into two groups (SOFA 0–1 and 2–4) using each organ's SOFA score components. We examined the odds ratio of death with and without PMX in each group.

### Statistical analysis

We reported continuous variables as the median and interquartile range (IQR) and categorical variables as number and percentage. We performed statistical analysis using JMP Pro 15.2.0 (SAS Institute Inc.) and used logistic analysis for multivariate analysis. We used the Kaplan–Meier method to compare survival curves, and χ^2^ test (Pearson method for *p-*value) to compare two groups for mortality within 28 days, and the Wilcoxon rank-sum test to compare free days.

## Results

### Patient selection

**Fig. 1:**
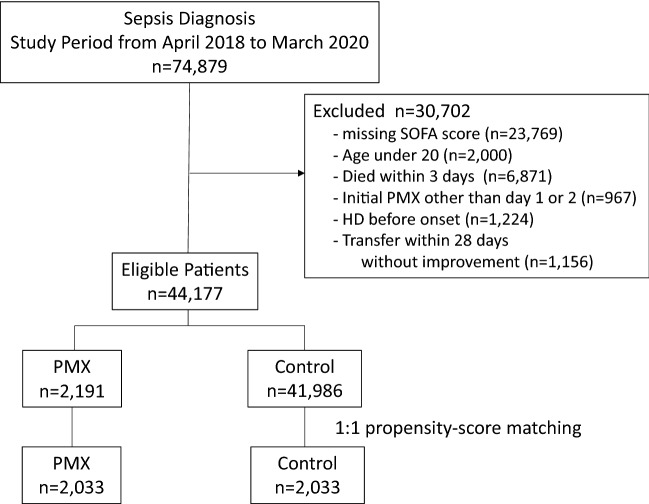
Patient selection flow

During the study period, 74,879 patients met the inclusion criteria of sepsis diagnosis. We excluded 30,702 patients and included 44,177 patients in the study. Missing SOFA scores were the most common reason for exclusion. Among the included patients, 2191 received PMX treatment, and 41,986 did not. After the 1:1 propensity score matching, we created a pair of 2033 patients (Fig. 1).

### Multivariate analysis

Table 1 shows the 28-day mortality odds ratio for each factor in the multivariate logistic regression analysis. Male, higher age, use of CHDF, H.D., mechanical ventilation, steroids, red blood cell transfusion, platelet transfusion, maximum noradrenaline dose, higher SOFA score, and higher CCI were the factors that increased mortality. Age, maximum noradrenaline dose (mg/day), SOFA score, and CCI were the odds ratios per unit, with SOFA score having a significant effect. On the other hand, admission to emergency rooms or intensive care units, university hospitals, use of PMX, surgery before the onset of sepsis, use of γ-globulin, and use of rTM were associated with lower mortality.

**Table 1:** 28-day mortality odds ratio in the multivariate logistic regression analysis

Variable	Odds ratio	95% CI		*p*-value
Age	1.033	1.030	1.035	< 0.0001
Sex (male)	1.187	1.118	1.260	< 0.0001
Emergency admission	0.720	0.626	0.828	< 0.0001
University hospital	0.737	0.666	0.814	< 0.0001
PMX	0.811	0.708	0.930	0.0027
CHDF	1.348	1.214	1.496	< 0.0001
HD	1.238	1.054	1.454	0.0092
Mechanical ventilation	1.441	1.319	1.575	< 0.0001
Surgery	0.482	0.434	0.535	< 0.0001
ER/ICU admission	0.789	0.733	0.849	< 0.0001
γ-Globulin	0.851	0.758	0.956	0.0066
rTM	0.947	0.848	1.058	0.3357
AT III	0.891	0.769	1.033	0.1263
Steroid	1.423	1.320	1.535	< 0.0001
RBC transfusion	1.868	1.704	2.048	< 0.0001
Platelet transfusion	1.212	1.066	1.377	0.0032
Max noradrenaline	1.006	1.003	1.010	0.0003
SOFA score	1.160	1.151	1.170	< 0.0001
CCI	1.082	1.064	1.100	< 0.0001

*PMX* polymyxin B hemoperfusion, *CHDF* continuous hemodiafiltration, *HD* hemodialysis, *ER* emergency room, *ICU* intensive care unit, *rTM* recombinant thrombomodulin, *AT* antithrombin, *RBC* red blood cell, *SOFA* Sequential Organ Failure Assessment, *CCI* Charlson Comorbidity Index

### Propensity score matching

Next, we performed a propensity score matching to compare the outcomes of patients between the PMX group and the control group. Table 2 shows the difference of each covariate before and after the matching. After the matching, ASD of all covariates was within 10%, showing the background characteristics of the two groups were well balanced. We did not include the SOFA score for propensity score calculation since we planned to compare two groups after stratifying based on the SOFA score. Before the matching, the PMX group was distributed in higher SOFA score categories than the control group, showing that baseline organ dysfunction was severe in the PMX group. After the matching, the distribution of SOFA score categories was well balanced (Table 3).

**Table 2:** Baseline patient characteristics before and after propensity score matching

Variable	Unmatched groups			Matched groups		
	PMX (*n* = 2191)	Control (*n* = 41,986)	ASD (%)	PMX (*n* = 2033)	Control (*n* = 2033)	ASD (%)
Age						
≦ 50	167 (7.6)	2477 (5.9)	5.7	159 (7.8)	144 (7.1)	2.3
51–70	640 (29.2)	9,048 (21.6)	14.7	593 (29.2)	674 (33.2)	7.0
> 70	1384 (63.1)	30,461 (16.8)	16.8	1281 (63.0)	1215 (59.8)	5.4
Sex (male)	1282 (58.5)	22,234 (9.1)	9.1	1183 (58.2)	1231 (60.6)	3.9
Emergency admission	1963 (89.6)	40,447 (23.5)	23.5	1835 (90.3)	1839 (90.5)	0.5
CCI						
0	529 (24.1)	9,798 (23.3)	1.6	492 (24.2)	488 (24.0)	0.4
1	457 (20.9)	10,388 (24.7)	7.5	420 (20.7)	421 (20.7)	0.1
2	498 (22.7)	9,383 (22.3)	0.7	460 (22.6)	441 (21.7)	1.8
≧ 3	707 (32.3)	12,417 (29.6)	4.8	661 (32.5)	683 (33.6)	1.9
University hospital	490 (22.4)	4383 (10.4)	28.1	449 (22.1)	496 (24.4)	4.4
Max noradrenaline						
< 5	483 (22.0)	30,812 (73.4)	96.8	476 (23.4)	416 (20.5)	5.9
5–9.9	402 (18.3)	4292 (10.2)	19.9	372 (18.3)	384 (18.9)	1.2
10–15.9	577 (26.3)	3691 (8.8)	41.7	528 (26.0)	560 (27.5)	2.9
≧16	729 (33.3)	3191 (7.6)	60.3	657 (32.3)	673 (33.1)	1.4
CHDF	1440 (65.7)	2715 ( 6.5)	142.4	1284 (63.2)	1297 (63.8)	1.1
HD	94 (4.3)	1119 (2.7)	7.5	90 (4.4)	118 (5.8)	5.0
Mechanical ventilation	1471 (67.1)	5430 (12.9)	114.8	1326 (65.2)	1352 (66.5)	2.2
Surgery	1200 (54.8)	3912 (9.3)	99.6	1048 (51.5)	1024 (50.4)	1.9
ER/ICU admission	1596 (72.8)	12,260 (29.2)	78.9	1469 (72.3)	1573 (77.4)	9.7
γ-Globulin	718 (32.8)	2417 (5.8)	66.6	620 (30.5)	593 (29.2)	2.4
rTM	1091 (49.8)	2353 (5.6)	104.8	946 (46.5)	901 (44.3)	3.6
AT III	538 (24.6)	1078 (2.6)	64.1	455 (22.4)	433 (21.3)	2.1
Steroid	1023 (46.7)	6847 (16.3)	59.5	941 (46.3)	996 (49.0)	4.4
RBC transfusion	881 (40.2)	3301 (7.9)	73.7	785 (38.6)	774 (38.1)	0.9
Platelet transfusion	424 (19.4)	1336 (3.2)	49.0	380 (18.7)	387 (19.0)	0.7

*CCI* Charlson Comorbidity Index, *CHDF* continuous hemodiafiltration, *HD* hemodialysis, *ER* emergency room, *ICU* intensive care unit, *rTM* recombinant thrombomodulin, *AT* antithrombin, *RBC* red blood cell

**Table 3:** The number of patients in each SOFA score category before and after propensity score matching

SOFA score	Unmatched groups			Matched groups		
	PMX (*n* = 2191)	Control (*n* = 41,986)	ASD (%)	PMX (*n* = 2033)	Control (*n* = 2033)	ASD (%)
0–6	483 (22.0)	27,006 (64.3)	75.3	456 (22.4)	407 (20.0)	4.8
7–9	580 (26.5)	7729 (18.4)	16.2	553 (27.2)	463 (22.8)	8.4
10–12	551 (25.1)	4499 (10.7)	33.1	510 (25.1)	529 (26.0)	1.7
13–15	399 (18.2)	1991 (4.7)	38.9	356 (17.5)	404 (19.9)	4.9
16–24	178 (8.1)	761 (1.8)	26.9	158 (7.8)	230 (11.3)	9.6

*SOFA* Sequential Organ Failure Assessment, *ASD* absolute standard difference

### Survival rate

**Fig. 2:**
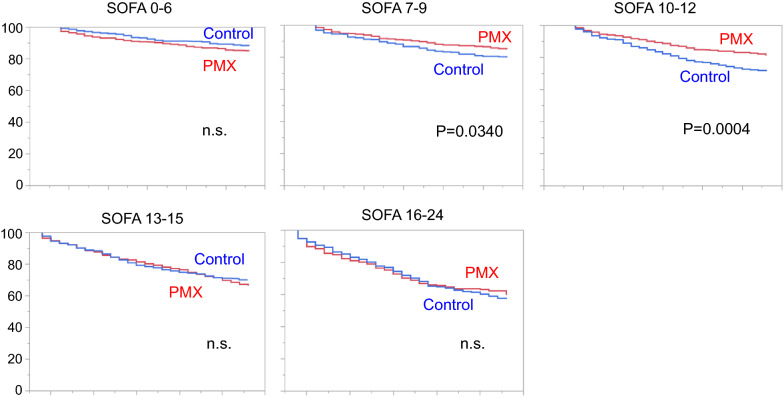
Kaplan–Meier survival plots for patients treated with or without PMX in propensity score-matched cohorts. Patients were stratified into five categories: SOFA score, 0–6, 7–9, 10–12, 13–15, and 16–24. Then, we compared the survival curves in each category

Figure 2 shows the Kaplan–Meier survival curves of each group, stratified by the SOFA score categories. In the SOFA score categories of 7–9 and 10–12, survival of the PMX group was significantly higher than the control group. On the other hand, there was no significant difference between the groups in the SOFA score categories of 0–6, 13–15, and 16–24. The 28-day mortality for the PMX and the control group was 15.0% and 19.9%, respectively, in the category of SOFA 7–9 (*p* = 0.0410), and 18.6% and 27.4%, respectively, in the category of SOFA 10–12 (*p* = 0.0008). These results confirm that PMX treatment is associated with a significant reduction in mortality in these ranges of baseline SOFA score. The detailed results of 28-day mortality analyses are provided in Additional file 1: Table S1.

### Organ support-free days

Table 4 shows the numbers of CHDF-, ventilator- and noradrenaline-free days at day 28. These analyses include patients who received each treatment on day one or day two. CHDF-free days were substantially longer in the PMX group in the SOFA score of 0–6, 7–9, and 10–12. Ventilator-free days were considerably longer in the PMX group in the SOFA score category of 7–9 and 10–12. Noradrenaline-free days were significantly longer in the PMX group in the SOFA score categories 7–9 and 10–12.

**Table 4:** CHDF-, ventilator- and noradrenaline-free days at day28

SOFA score	CHDF-free days					Ventilator-free days					Noradrenaline-free days				
	PMX		Control		*p*-value	PMX		Control		*p*-value	PMX		Control		*p*-value
	*n*	Median (IQR)	*n*	Median (IQR)		*n*	Median (IQR)	*n*	Median (IQR)		*n*	Median (IQR)	*n*	Median (IQR)	
0–6	250	24 (20–26)	169	23 (10.5–25)	0.0012	289	21 (4–25)	198	21 (5–25)	0.9257	338	25 (21–26)	278	25 (20–26)	0.9287
7–9	335	23 (15–25)	289	21 (0–24)	< 0.0001	356	20 (2–24)	294	17(0–23)	0.0118	484	25 (20–26)	390	24 (11–26)	0.0003
10–12	362	23 (8–25)	400	21 (0–24)	0.0034	382	16 (0–23)	412	12 (0–22)	0.0061	486	24 (15–26)	493	22 (0–26)	0.0005
13–15	281	20 (0–24)	334	19 (0–23)	0.8179	303	10 (0–22)	351	9 (0–21)	0.5397	345	22 (0–25)	390	21.5 (0–25)	0.9161
16–24	144	12.5 (0–22)	212	8 (0–22)	0.6808	145	1 (0–19)	215	1 (0–18)	0.6976	155	14 (0–24)	227	15 (0–24)	0.3491

*SOFA* Sequential Organ Failure Assessment, *IQR* interquartile range, *CHDF* continuous hemodiafiltration

### Stratification by the individual SOFA score components

**Fig. 3:**
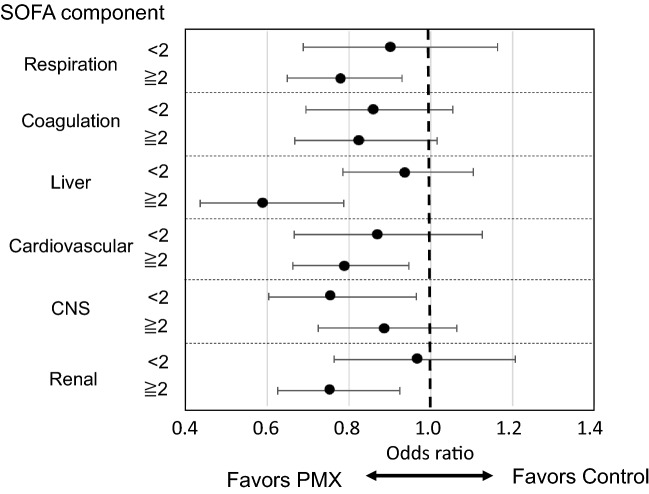
Odds ratio of 28-day mortality across subsets defined according to individual SOFA score components of each organ

Figure 3 shows the 28-day survival odds ratios between the PMX and control groups, stratified by each organ's SOFA score components. For respiration, coagulation, liver, cardiovascular and renal SOFA, the odds ratio of death in the PMX group tended to be lower in the high SOFA score group (2 or more) than the low SOFA score group (less than 2). On the other hand, the odds ratio of death tended to be higher in the high SOFA score group than the low SOFA score group for the central nervous system SOFA.

## Discussion

In this study, we examined the association between the severity of organ failure and the efficacy of PMX by using the Japanese nationwide inpatient database, the DPC data. After adjusting the patient background characteristics by propensity score matching, we found that PMX significantly improves the survival of sepsis patients in the SOFA score ranges of 7–9 and 10–12. On the other hand, there was no significant difference in the survival rate in SOFA score ranges of 0–6, 13–15, and 16–24. In analyzing organ support-free days, PMX was also effective in the SOFA ranges of 7–9 and 10–12, compared to 0–6, 13–15, and 16–24. In a more detailed analysis comparing the mortality difference in each SOFA score, the risk ratio of 28-day morality was lower than 1 in SOFA 6, 7, 8, 9, 12, 14, 16, 17, 20, and ≧ 21. In addition, the survival benefit was statistically significant in SOFA 9, 10, 12 (Additional file 1: Table S1). The cut-off of the SOFA score in which PMX is effective cannot be strictly determined. However, as a rough guide, PMX is considered effective in patients with moderate disease severity in the range of 7 to 12.

Several previous studies have examined the effectiveness of PMX using the DPC data. In one study focused on septic shock due to lower gastrointestinal perforation, the 28-day mortality rates for patients with and without PMX were 17.1% and 16.3%, respectively, which were not significantly different [14]. Conversely, in a study on septic shock with AKI who required CRRT, the 28-day mortality rates with and without PMX were 40.2% and 46.7%, respectively, with a significant improvement with PMX treatment [15]. Our recent study on noradrenaline-treated septic shock patients showed a substantial improvement of 28-day survival rates by PMX treatment, 77.9% for the PMX group and 71.1% for the non-PMX group [16]. In addition, our analysis of data from patients with sepsis requiring CHDF showed that PMX significantly improved mortality and shortened hospital and ICU stays [17]. All studies used the propensity score matching technique to adjust the patient background between PMX-treated and non-treated groups. However, none of the studies used the SOFA score as an adjustment factor of patient background or a subgroup stratification factor since the database did not include the SOFA score before March 2018.

This study, which used the DPC data after April 2018, is the first to utilize the SOFA score recorded in the DPC database to analyze the efficacy of sepsis treatment. The SOFA score, which reflects the degree of damage to multiple organs, has been widely used as a factor reflecting the severity of sepsis in patients. Many studies have reported that it is highly associated with mortality. Using the SOFA scores is a powerful method for analyzing large-scale registry data such as DPC and examining the effectiveness of various treatments for critically ill patients in actual clinical practice in detail.

Several multicenter RCTs have been conducted to evaluate the efficacy of PMX on septic shock. However, they showed inconsistent findings regarding the survival benefit. We assume that one reason for the lack of apparent efficacy of PMX in those RCTs may be that the included patients were heterogeneous with varying severity of the disease. In the EUPHRATES trial, the largest RCT of PMX conducted so far, the analysis of all enrolled patients showed no difference in the mortality between the PMX and control groups [9]. But a post hoc study showed a significant benefit of PMX in the patients with Multiple Organ Dysfunction Scores (MODS) of 10 or more and endotoxin activity assay (EAA) levels in the range of 0.6 to 0.9 [18]. A new randomized controlled trial targeting this specific cohort is ongoing in the U.S. [19]. Although EAA is a measurement of endotoxin levels, it is also considered a marker that reflects the degree of organ damage [20]. Thus, the post hoc analysis of the EUPHRATES trial is consistent with the results of this study using the SOFA score. The research confirmed that PMX is the most effective in patients with an intermediate range of organ damage.

Our previous study on the analysis of noradrenaline-administered septic shock patients showed that PMX efficacy is less pronounced in the subgroup of patients with the highest maximum daily dose of noradrenaline [16]. Furthermore, several reports indicate that PMX treatment is more effective when the time between the onset of shock and the administration of PMX is shorter [21, 22]. Therefore, the present analysis results using the SOFA score suggest that it is vital to use PMX before organ damage progresses too far.

In the stratified analysis using individual SOFA score components of each organ, the survival benefit of PMX tended to be higher in patients with central nervous system SOFA scores of less than two than in patients with scores of two or more. On the other hand, the effect tended to be higher in patients with scores of 2 or more for respiration, coagulation, liver, cardiovascular, and renal SOFA score. This result suggests that optimal timing may vary depending on the type of organ that is impaired. However, the present analysis did not consider the correlation between each organ damage. Thus, further study is needed to determine the impacts of individual organ damage on the efficacy of PMX.

One study aimed to identify the optimal population for PMX using the sepsis database, which could not show the correlation between PMX efficacy and SOFA score [23]. However, only 92 patients received PMX in that study. Therefore, it may not have enough power to analyze the precise relationship of PMX to various patient conditions. The strength of our research is that we analyzed data containing more than 2000 cases of PMX treatment, which enabled more in-depth analysis.

The SOFA score is widely used to indicate of organ damage in critically ill patients. There are many reports on the association of SOFA score and the prognosis of sepsis patients [24, 25]. The latest definition of sepsis, Sepsis-3, also uses an increase in SOFA score to indicate organ damage [26]. The SOFA score is relatively easy and quick to obtain. It is an item routinely assessed in daily clinical settings. This study provides helpful information for the selection of suitable patients for PMX treatment in real-world clinical settings.

This study has several limitations. First, the study is a retrospective analysis of data. Although we adjusted for possible background factors by propensity score matching, we cannot rule out the presence of confounding factors. These include vital signs or laboratory data, which are not available in the DPC database. Second, the disease code of sepsis is based on clinical judgment and not always based on the international definition of Sepsis-3.

## Conclusion

Analysis of a large-scale Japanese inpatient database found a significant association between PMX efficacy and baseline SOFA score. These findings suggest higher efficacy in patients with medium SOFA scores in the range of 7–12. The result provides a promising hypothesis for selecting appropriate patients for PMX and should be validated in future RCTs.

## Supplementary Information

**Additional file 1: Table S1.** Detailed analysis of 28-day-mortality differences between PMX group and control group in each single SOFA score.

## Data Availability

Not applicable.

## Abbreviations

PMX: Polymyxin B hemoperfusion
SOFA: Sequential Organ Failure Assessment
CHDF: Continuous hemodiafiltration
DPC: Diagnosis Procedure Combination
E.R.: Emergency room
ICU: Intensive care unit
RCT: Randomized controlled trial
CCI: Charlson Comorbidity Index
H.D.: Hemodialysis
AT: Antithrombin
rTM: Recombinant thrombomodulin
RBC: Red blood cell
ASD: Absolute standard difference
IQR: Interquartile range
MODS: Multiple Organ Dysfunction Score
