# Supplementary material for: Effectiveness of polymyxin B hemoperfusion for sepsis depends on the baseline SOFA score: a nationwide observational study
Source: Ann Intensive Care. 2021 Sep 26;11:141. doi: 10.1186/s13613-021-00928-z (PMC8473472; doi:10.1186/s13613-021-00928-z)
Supplement: Supplementary file 1 — Additional file 1: Table S1. Detailed analysis of 28-day-mortality differences between PMX group and control group in each single SOFA score. [file 13613_2021_928_MOESM1_ESM.docx]

Table S1 Detailed analysis of 28-day-mortality differences between PMX group and control group in each single SOFA score.

| SOFA | The number of patients | | The number of dead patients | | 28 day-mortality (%) | | Risk ratio | p-value |
| --- | --- | --- | --- | --- | --- | --- | --- | --- |
|  | PMX(＋) | PMX(-) | PMX(＋) | PMX(-) | PMX(＋) | PMX(-) |  |  |
| ≦5 | 323 | 275 | 49 | 25 | 15.2% | 9.1% | 1.669 | 0.024 |
| 6 | 133 | 132 | 20 | 22 | 15.0% | 16.7% | 0.902 | 0.717 |
| 7 | 161 | 136 | 26 | 22 | 16.1% | 16.2% | 0.998 | 0.995 |
| 8 | 180 | 152 | 27 | 30 | 15.0% | 19.7% | 0.760 | 0.255 |
| 9 | 212 | 175 | 30 | 40 | 14.2% | 22.9% | 0.619 | 0.027 |
| 10 | 169 | 160 | 25 | 49 | 14.8% | 30.6% | 0.483 | 0.001 |
| 11 | 154 | 194 | 39 | 49 | 25.3% | 25.3% | 1.003 | 0.989 |
| 12 | 187 | 175 | 31 | 47 | 16.6% | 26.9% | 0.617 | 0.017 |
| 13 | 151 | 161 | 43 | 40 | 28.5% | 24.8% | 1.146 | 0.468 |
| 14 | 121 | 121 | 40 | 43 | 33.1% | 35.5% | 0.930 | 0.685 |
| 15 | 84 | 122 | 33 | 35 | 39.3% | 28.7% | 1.369 | 0.112 |
| 16 | 62 | 81 | 19 | 28 | 30.6% | 34.6% | 0.887 | 0.621 |
| 17 | 34 | 66 | 13 | 28 | 38.2% | 42.4% | 0.901 | 0.687 |
| 18 | 30 | 35 | 15 | 15 | 50.0% | 42.9% | 1.167 | 0.565 |
| 19 | 14 | 23 | 5 | 11 | 35.7% | 47.8% | 0.747 | 0.471 |
| 20 | 13 | 18 | 8 | 10 | 61.5% | 55.6% | 1.108 | 0.739 |
| ≧21 | 5 | 7 | 2 | 4 | 40.0% | 57.1% | 0.700 | 0.976 |
